# Dielectric Properties of Graphene/Titania/Polyvinylidene Fluoride (G/TiO_2_/PVDF) Nanocomposites

**DOI:** 10.3390/ma13010205

**Published:** 2020-01-03

**Authors:** Saira Ishaq, Farah Kanwal, Shahid Atiq, Mahmoud Moussa, Umar Azhar, Dusan Losic

**Affiliations:** 1 Institute of Chemistry, University of the Punjab, Lahore 54590, Pakistan; saira_chem@yahoo.com; 2 School of Chemical Engineering, The University of Adelaide, Adelaide, SA 5005, Australia; mahmoud.moussa@adelaide.edu.au (M.M.); umar_azhar@hotmail.com (U.A.); 3 The ARC Research Hub for Graphene Enabled Industry Transformation, The University of Adelaide, Adelaide, SA 5005, Australia; 4 Centre of Excellence in Solid State Physics, University of the Punjab, Lahore 54590, Pakistan; satiq.cssp@pu.edu.pk

**Keywords:** graphene, titania, polyvinylidene fluoride, nanocomposite, dielectric properties

## Abstract

Flexible electronics have gained eminent importance in recent years due to their high mechanical strength and resistance to environmental conditions, along with their effective energy storage and energy generating abilities. In this work, graphene/ceramic/polymer based flexible dielectric nanocomposites have been prepared and their dielectric properties were characterized. The composite was formulated by combining graphene with rutile and anatase titania, and polyvinylidene fluoride in different weight ratios to achieve optimized dielectric properties and flexibility. After preparation, composites were characterized for their morphologies, structures, functional groups, thermal stability and dielectric characterizations by using scanning electron microscopy, X-ray diffraction, Raman spectroscopy, Fourier transform infrared spectroscopy, thermal gravimetric analysis and impedance spectroscopy. Dielectric results showed that prepared flexible composite exhibited dielectric constant of 70.4 with minor leakage current (tanδ) i.e., 0.39 at 100 Hz. These results were further confirmed by calculating alternating current (AC) conductivity and electric modulus which ensured that prepared material is efficient dielectric material which may be employed in electronic industry for development of next generation flexible energy storage devices.

## 1. Introduction

Flexible electronics are attracting increased attention in the electronic industry day by day due to their compact nature and ease of handling. Along with other electronics, flexible dielectric materials are also getting popular for many applications like in field effect transistors (FET), gate dielectrics, pulse power systems, sensors and energy storage devices, etc [1,2,3,4]. Polymers are attractive to synthesize flexible materials but they have limitations of low capacitance and low dielectric constants [4]. In last few years efforts have been made to enhance dielectric constant (ε′) of polymers either by using dielectric or conducting fillers [5,6].

Conducting fillers can elevate ε′ to a much higher value, but the amount of conducting filler plays a critical role in this increase. The dielectric response of conducting filler/polymer composites depends upon the percolation phenomenon. In such composites if the concentration of conducting filler is lower than its percolation threshold (*ϕ_c_*), an insulative-dielectric environment exists. However with the increase in concentration of conducting fillers, ε′ increases and reaches a maximum limit when *ϕ_c_* is reached, after which it starts decreasing [7]. A number of composites which consist of conducting fillers and polymers have been reported, but a high ε′ is also associated with high energy losses in the case of conducting fillers. Feng et al. [8] reported a silver/poly(vinylidene fluoride) (Ag/PVDF) composite exhibiting ε′ of 31 and loss tangent (tanδ) of 0.02 at 1 kHz. Zhao and group [9] synthesized a carbon nanotubes/poly(vinylidene fluoride) (CNT/PVDF) composite with ε′ of 80.6 and tanδ of 3.51 at 100 Hz. They also reported ε′ of 112.1 and low tanδ of 0.032 for graphene nanoplatelet/PVDF in the same paper.

Dielectric fillers increase ε′ when added to polymers. However, in this case the final dielectric properties depend upon many factors like size, shape, distribution, concentration and connectivity of dielectric fillers in the polymer [7]. Pan et al., [10] used barium titanate nanotubes modified with dopamine in poly(vinylidene fluoride (PVDF), and the resultant composite had ε′ of 47.05 at 1 kHz with very low dielectric loss (ε″). Yang et al. reported that nanosize calcium copper titanate particles are more effective in enhancing ε′ than their microsize equivalents. Nanosize particles increased ε′ of PVDF from 10.5 to 2.49 × 10^6^ at 100 Hz with tanδ of 48. However, the microsize version resulted in ε′ of 49 with tanδ of 0.23 at 100 Hz [11]. The ε′ of such composites is increased with an increase in filler content but when such fillers are added in excessive amount, the flexibility and mechanical strength of the composite is decreased [12]. Another approach is to incorporate conducting and ceramic fillers simultaneously in the polymer matrix. Wang et al., [13] synthesized a copper calcium titanate/PPO composite with ε′ of 7.08 which was increased when copper calcium titanate was coated with graphene and then its composite was made with poly(phenylene oxide) (PPO). The resultant composite had ε′ of 8.60 and tanδ of 0.017 at 100 Hz. Qi and coworkers [14] reported ternary composites consisting of carbon nanotubes loaded with barium titanate/polyamide11. The calculated ε′ of the composite at 1 kHz was 16.2. Also they measured tanδ of the same composite at 100 Hz and it was 0.15. Wan and group reported synthesis of a ternary composite based on functionalized graphene/barium titanate/ polyvinylidene fluoride (frGO/BaTiO_3_/PVDF) which has ε′ of 65 at 1 MHz and tanδ of 0.35 at same frequency [15].

The aim of present research work was the preparation of graphene/titania/polyvinylidene fluoride (G/TiO_2_/PVDF) composite films with different dosages and ratios of their components and to investigate their influence on dielectric properties. To formulate these three phase composites, we selected every component considering their specific properties and entire properties of the final composite. The PVDF is semicrystalline in nature, having piezoelectric characteristics, high ε′ and tanδ. These characteristics render it most appropriate candidate for dielectric applications [16]. Graphene was used as rational filler to exalt properties of composites as it elevates high mechanical strength, electrical properties and reasonable thermal stability originating from oxygen functional groups on its surface [17]. Among ceramics, TiO_2_ was selected because of its semiconductor nature and optical properties. Due to its electrical properties it has replaced silica in dielectric devices. It exists in three crystalline forms viz; brooklite, anatase and rutile. Among them anatase and rutile forms are reported to possess dielectric properties [18]. In the present research work, mixture of anatase and rutile forms of TiO_2_ was used. Dielectric analysis of the composites confirmed excellent dielectric properties of the prepared composites that render them as effective flexible dielectric materials.

## 2. Materials and Methods

### 2.1. Materials

Commercial graphite rocks were purchased from Uley (Eyre Peninsula, SA, Australia). Polyvinylidenefluroride pellets and potassium permanganate (99%) were bought from Sigma-Aldrich (Gillman, Australia). Hydrochloric acid (37%), hydrogen peroxide (35%), sulfuric acid and N,N–dimethylformamide were purchased from Chem-supply (Gillman, Australia). Potassium persulphate (K_2_S_2_O_8_) was purchased from Scharlau (Germany). Phosphorus pentoxide (P_2_O_5_) and titanium(IV) oxide (purity > 99.5% having specific surface area of 9.6 m^2^ g^−1^) were taken from Riedel-de Haen (Germany). All reagents were used without any treatment and purification.

### 2.2. Preparation of Graphene/Titania/Polyvinylidene Fluoride Nanocomposite Films

**Figure 1:**
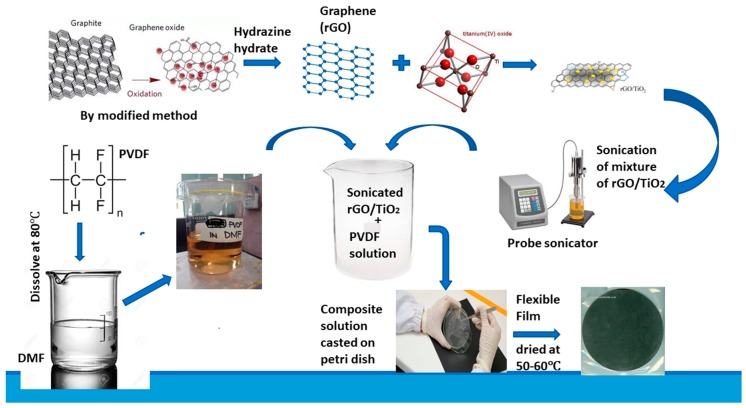
Scheme of preparation of three phase graphene/titania/polyvinylidene fluoride nanocomposite films.

Graphite powder was oxidized to graphene oxide (GO) by a previously reported method [19]. Graphene was synthesized by reduction of GO by using hydrazine hydrate [20]. Firstly, 1 g of PVDF was completely mixed in N,N–dimethylforamide (DMF, 20 mL) while heating and stirring at 80 °C. Specific amounts of TiO_2_ and graphene were dispersed separately in 10 mL of DMF by using a probe sonicator (Branson Digital Sonifier 450, Branson Ultrasonics Corporation, Danbury, CT, USA). Both solutions were mixed while stirring at 80 °C for 45 min. Film of G/TiO_2_/PVDF composite was casted on petri dish by drying the solution at 60 °C. Firstly few composite films of G/TiO_2_/PVDF were prepared by varying amounts of graphene while amounts of other components were kept constant. After measuring their ε′, graphene concentration giving maximum ε′ was fixed while few more films were fabricated with varying amounts of TiO_2_. G_x_T_y_PVDF, G_2x_T_y_PVDF, G_3x_T_y_PVDF, G_4x_T_y_PVDF, G_5x_T_y_PVDF, G_4x_T_2y_PVDF and G_4x_T_3y_PVDF (where x = 0.01, y = 0.1) were symbolized as G1T10PVDF, G2T10PVDF, G3T10PVDF-, G4T10PVDF, G5T10PVDF, G4T20PVDF and G4T30PVDF, respectively. Amount of PVDF in all samples was fixed i.e., 1 g. A scheme of the preparation of the films is shown in Figure 1.

### 2.3. Characterizations

Pure PVDF and ternary nanocomposites films of size 0.5 cm × 0.5 cm (width × length) were characterized by field emission scanning electron microscopy (FESEM, Quanta 450, FEI, Hillsboro, OR, USA), X-ray diffraction (XRD, 600 Miniflex, Rigaku, Akishima, Japan) (sample film size: width × length = 1.5 cm × 1.5 cm) raman spectroscopy (LabRam HR Evolution, Horiba-Yvon Jobin Technology, Kyoto, Japan) (sample size: width × length = 0.5 cm × 0.5 cm) and Fourier transform infrared (FTIR) spectroscopy (Nicolet 6700, Thermo Fisher, Waltham, MA, USA) (sample size: width × length = 0.5 cm × 0.5 cm). Thermal gravimetric analyzer (TGA, Q500, TA Instruments, New Castle, DE, USA) for 7 mg of each sample was operated at 30–900 °C at heating rate of 10 °C min^−1^ A precision impedance analyzer (6500B, Wayer Kerr, West Sussex, UK) was used at 20 Hz–2 MHz.

## 3. Results and Discussion

### 3.1. Characterization of Graphene/Titania/Polyvinylidene Fluoride Nanocomposite Films

**Figure 2:**
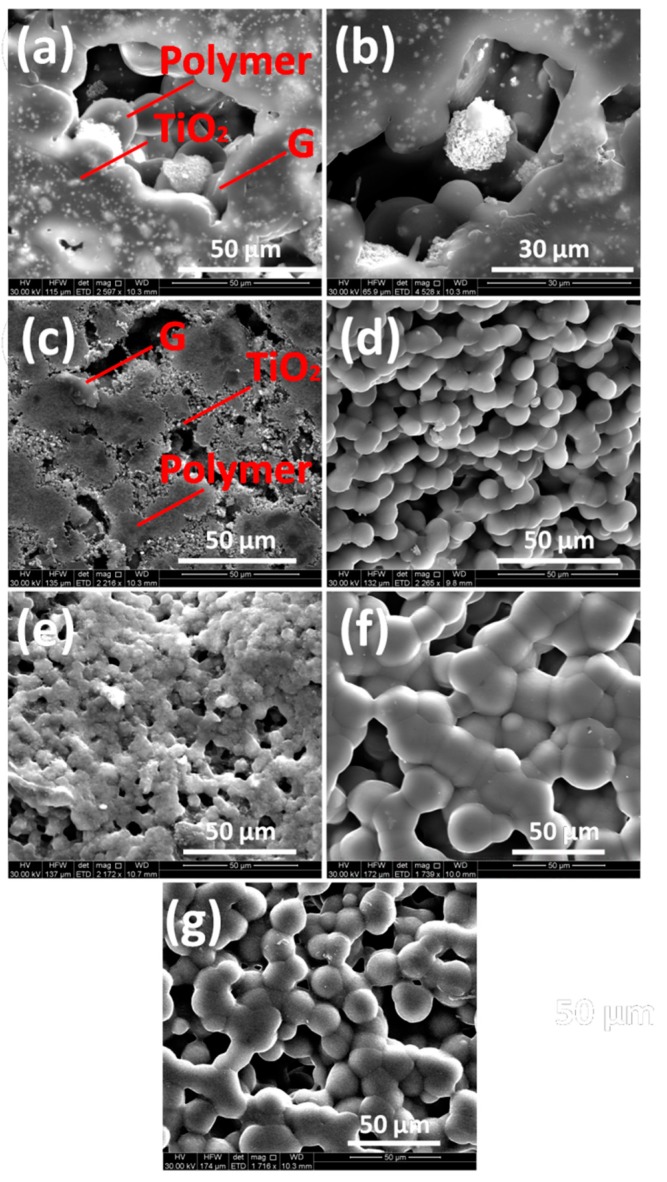
FESEM images of (**a**) G1T10PVDF (**b**) G2T10PVDF (**c**) G3T10PVDF (**d**) G4T10PVDF (**e**) G5T10PVDF (**f**) G4T20PVDF (**g**) G4T30PVDF.

The FESEM images of nanocomposites shown in Figure 2 were recorded to study their morphology. In the FESEM images graphene sheets, TiO_2_ particles and PVDF are clearly visible. Both fillers are evenly distributed in the polymer. It is evident that graphene sheets are segregated rather than being aligned due to occurrence of TiO_2_ and PVDF. Nanoparticles of TiO_2_ lie on graphene layers and the polymer. With increase in weight of TiO_2_, big aggregates start to form which affect dielectric properties of the nanocomposites which would be discussed in detail later in dielectric plots.

**Figure 3:**
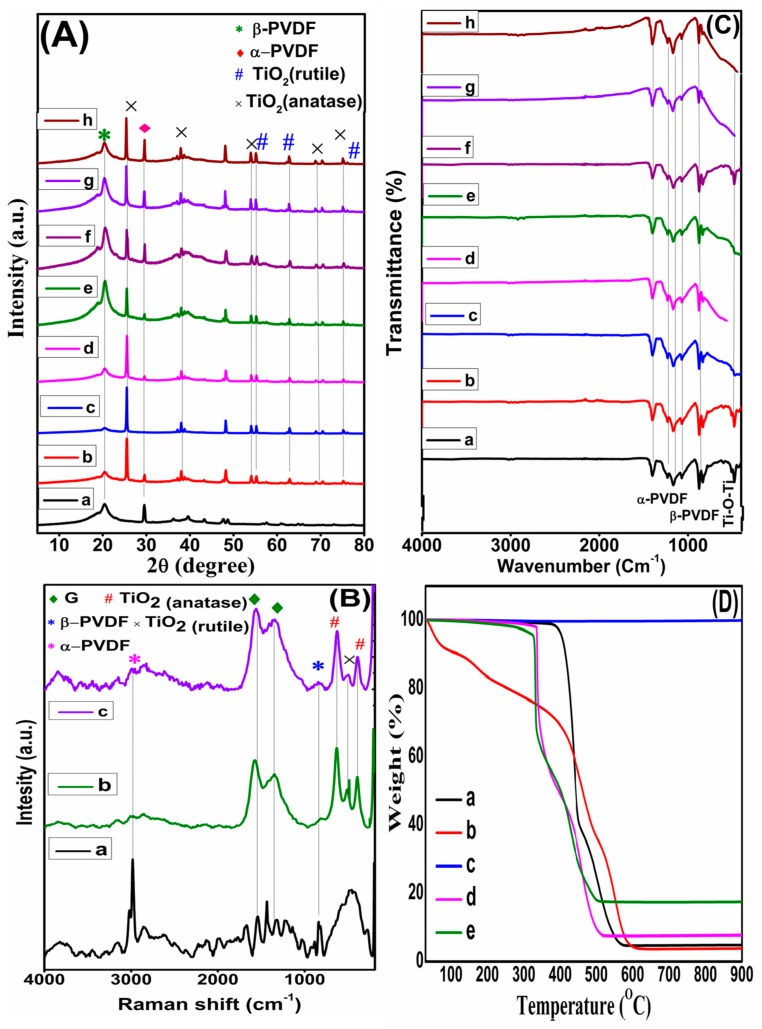
(**A**) XRD patterns (**a**) PVDF (**b**) G1T10PVDF (**c**) G2T10PVDF (**d**) G3T10PVDF (**e**) G4T10PVDF (**f**) G5T10PVDF (**g**) G4T20PVDF (**h**) G4T30PVDF (**B**) Raman spectra of (**a**) PVDF (**b**) G4T10PVDF (**c**) G4T20PVDF (**C**) FTIR spectra of (**a**) PVDF (**b**) G1T10PVDF (**c**) G2T10PVDF (**d**) G3T10PVDF (**e**) G4T10PVDF (**f**) G5T10PVDF (**g**) G4T20PVDF (**h**) G4T30PVDF (**D**) TG curves of (**a**) PVDF (**b**) graphene (**c**) TiO_2_ (**d**) G4T10PVDF (**e**) G4T20PVDF.

XRD patterns of neat polymer and nanocomposite are shown in Figure 3A. In XRD pattern of pure PVDF, peaks at 20.4° and 29.7° are indicative of the beta (β) and alpha (α) phases of PVDF, respectively [21]. In XRD patterns of nanocomposites, the peaks located at 25.7°, 37.9°, 54.1°, 69.1° and 75.3° correspond to (101), (004), (211), (220) and (215) are diffraction peaks of anatase TiO_2_. These peaks are exactly according to JCPDS No. 21-1272. X-ray diffraction peaks of rutile TiO_2_ according to JCPDS No. 21-1276 are observed at 55.3° (220), 62.8° (200), and 70.5° (112) [22]. Intensity of peak of α-PVDF at 29.7° decreases due to interaction of fillers with PVDF. Peak for graphene is not visible in XRD peaks of nanocomposites. It may be due to presence of peak for anatase TiO_2_ which lie at 25.7° and also due to the reason that graphene is exfoliated and segregated during sonication process. This isolation of graphene layers is also shown in FESEM images in Figure 2. Raman spectra further elucidating the structure of pure PVDF and two selected nanocomposites i.e., G4T10PVDF and G4T20PVDF are shown in Figure 3B.

The band at 839 cm^−1^ is indicative of β-PVDF. Bands at 397 and 630 cm^−1^ in the composites correspond to anatase TiO_2_. Bands at 1352 and 1338 cm^−1^ in nanocomposites belong to D band and 1574 cm^−1^ correspond to G band of graphene [21,23].

FTIR spectra of pure polymer and it’s all synthesized nanocomposite are shown in Figure 3C. Peaks at 1077, 1156 and 1406 cm^−1^ confirm α-PVDF. Peaks at 827, 879 and 1234 cm^−1^ show presence of β-PVDF [24]. Peaks for graphene at 1077 and 1156 cm^−1^ are merged with peaks of PVDF. N–H stretching peak at 1406 cm^–1^ is due to DMF residues in the nanocomposites [21]. Peak for stretching vibrations of Ti-O-Ti bonds of anatase TiO_2_ is visible at 472 cm^−1^ [25]. TG curves of graphene, TiO_2_, pure polymer, and two selected samples i.e., G4T10PVDF and G4T20PVDF nanocomposites recoded at 30–900 °C are shown in Figure 3D. TG curve of graphene show that degradation of graphene starts at 380 °C and at 590 °C it completely degrades. TiO_2_ does not degrade even at high temperature of 900 °C. At 380 °C degradation of PVDF starts and at 565 °C, 96% of it degrades. G4T10PVDF and G4T20PVDF starts to degrade at 327 °C and 334 °C, and lose about 93% and 82% of their weights at 510 °C and 500 °C, respectively. More TiO_2_ in G4T20PDMS is responsible for increased thermally stability.

### 3.2. Dielectric Studies of Graphene/Titania/Polyvinylidene Fluoride Nanocomposite Films

**Figure 4:**
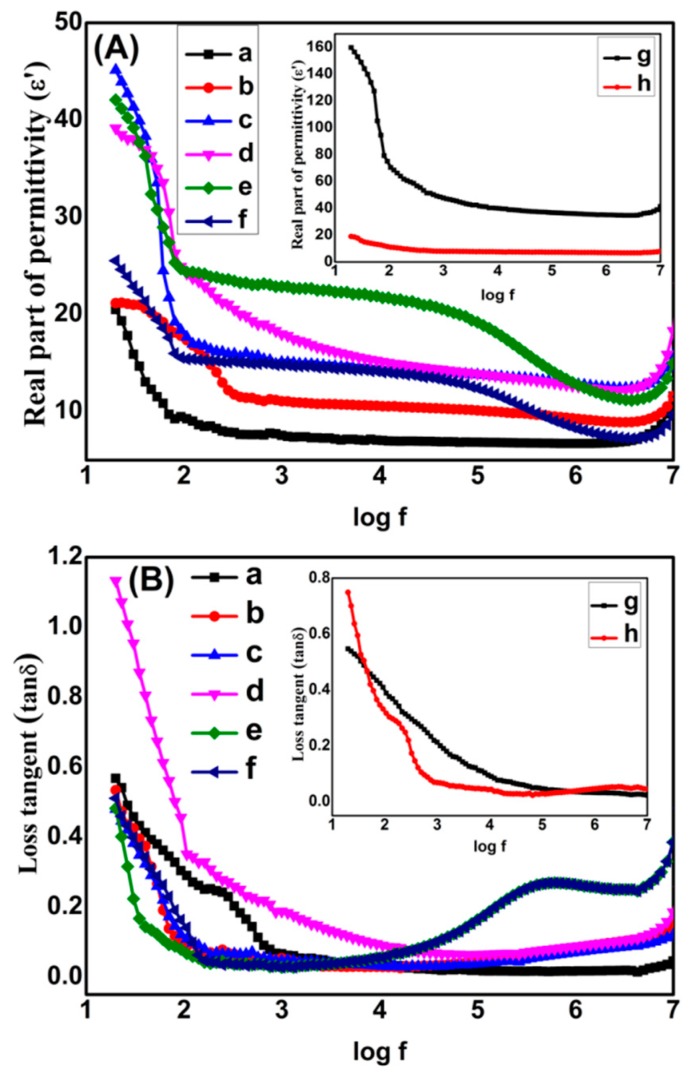
(**A**) Dielectric constant (ε′) (**B**) loss tangent (tanδ) of (**a**) PVDF (**b**) G1T10PVDF (**c**) G2T10PVDF (**d**) G3T10PVDF (**e**) G4T10PVDF (**f**) G5T10PVDF (**g**) G4T20PVDF (**h**) G4T30PVDF.

A Wayer Kerr 6500B precision impedance analyzer was used at 20 Hz–2 MHz to study the dielectric properties of the samples. Films were placed between copper electrodes and measurements were made. Dielectric constant of films was measured by using relation; ε′ = Ct/ɛ_o_A where C, t, ε_o_ and *A* represent capacitance, thickness of film, vaccum permittivity and area of the film. ε′ vs log f plots of pure PVDF and G/TiO_2_/PVDF nanocomposites are shown in Figure 4A. ε′ is high at lower frequencies due to fact that induced and permanent dipoles can accommodate themselves in proper alignment in this region, a phenomenon called the Maxwell Wagner Sillar (MWS) effect, also termed as interfacial polarization (IP). However, when frequency is increased, dipoles have less time to line up themselves in proper orientations, thus decreasing MWS and IP. This process is called polarization relaxation phenomenon [26]. At very high frequency range, ε′ becomes constant being frequency independent, a phenomenon called microcapacitor structure model [27]. ε′ increases when w/w of graphene is raised till G4T10PVDF which has value of 42.1 at 20 Hz. ε′ decreases with rising frequency and reaches and maintains to 11.6 at 2 MHz. But still it is greater than that of pure PVDF. Increase of ε′ with amount of graphene is due to reason that graphene is conductive and when its amount is increased it causes rise of IP. As conducting graphene is increased in the nanocomposite, more and more free charges accumulate at interfaces of the components thus forming more dipoles. Both increased interfacial area and homogeneous distribution of fillers contribute in rise of IP [26,28]. It was reported earlier that when conducting and non-conducting additives are embedded in the composites simultaneously, microcapacitors are formed where conducting part act as electrodes and non-conducting part acts as dielectric medium. In presently prepared nanocomposites, graphene sheets acts as electrodes while ceramic and polymers act as dielectric medium of the microcapacitors. As the amount of graphene is increased the number of microcapacitors also multiplies till G4T10PVDF. However after G4T10PVDF when the graphene concentration is further raised in G5T10PVDF, ε′ starts to decline. It is due to the fact that graphene sheets become associated to make flow of charges possible, thus the leakage current is raised to depress ε′ [29]. To further enhance ε′ of the nanocomposites the amount of ceramic was raised by keeping all other concentrations constant. ε′ again raised till the percolation level of ceramic was reached in G4T20PVDF. G4T20PVDF possesses highest ε′ among all nanocomposites i.e., 159.8 at 20 Hz which declined and was maintained to 34.8 at 2 MHz. The rise in ε′ after increasing amounts of ceramic is attributed to expansion of effective charge transport network to improve the conductivity within the nanocomposites [30,31]. However after reaching the percolation level when the amount of ceramic was further raised ε′ was decreased as rising amounts of ceramic result in aggregation of ceramic nanoparticles which not only effects the morphology, but also the efficiency of the charge transport network and the dielectric properties as well.

**Figure 5:**
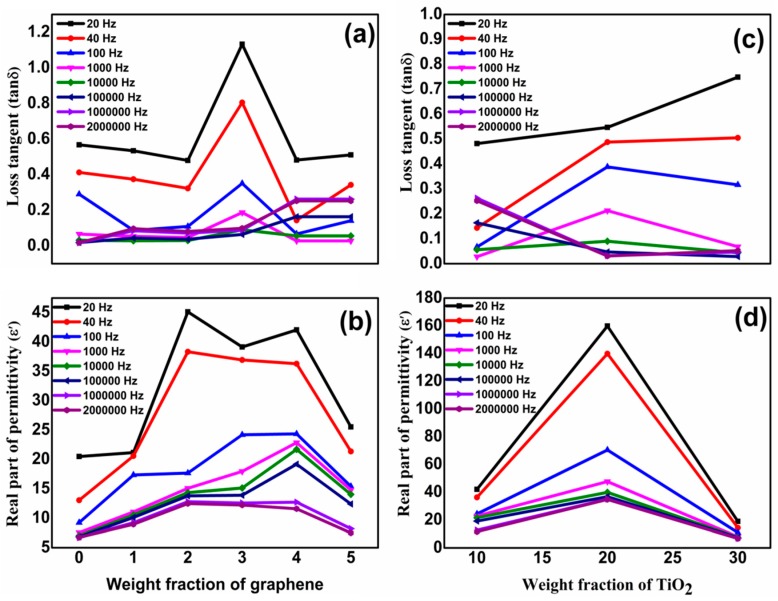
(**a**) Loss tangent (tanδ) (**b**) Real part of permittivity of neat PVDF and G/TiO_2_/PVDF nanocomposites films with different graphene fraction (**c**) Loss tangent (tanδ) (**d**) Real part of permittivity of G/TiO_2_/PVDF nanocomposites films with different TiO_2_ fraction.

Energy dissipation of pure polymer and all nanocomposites was calculated in the form of tanδ by using formula: Tanδ = ε″/ε′ where ε″ and ε′ represent the dielectric loss and dielectric constant, respectively. Plots of tanδ of PVDF and all nanocomposites against frequency are shown in Figure 4B. It is clear that tanδ decreases with raised frequency. The effect of different amounts of fillers on tanδ was also studied. It was found that when the amount of graphene is increased, tanδ also increases as well aligned conducting graphene sheets cause an increase of the leakage current density. Upon application of an electrical field current starts to flow through the conducting graphene as result of which electrical energy is converted into thermal energy [29]. At 20 Hz, tanδ of G4T10PVDF is 0.5 which reduces to 0.25 at 2 MHz. Tanδ decreases even more with addition of TiO_2_ and G4T20PVDF has tanδ of 0.5 at 20 Hz which further reduces o 0.03 at 2 MHz. Comparative plots of ε′ and tanδ of pure polymer and nanocomposites at various frequencies are shown in Figure 5a–d.

**Figure 6:**
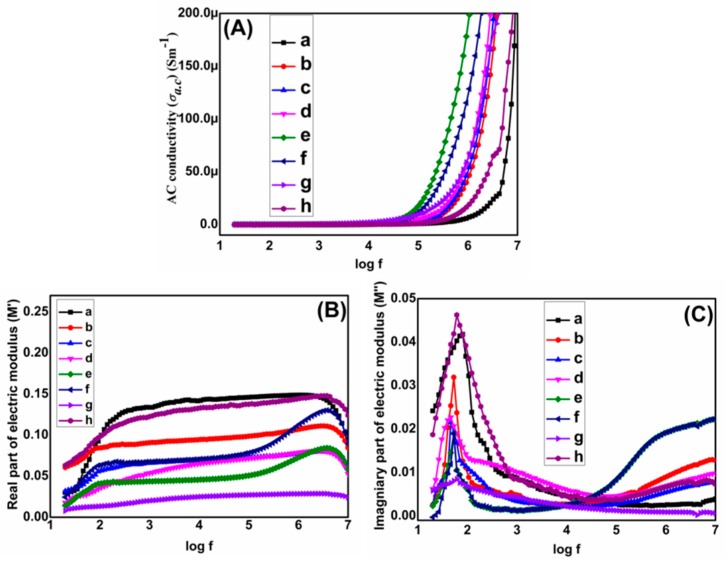
(**A**) AC conductivity (σ*_ac_*) (**B**) Real part of electric modulus (M′) (**C**) Imaginary part of electric modulus (M″) of (**a**) PVDF (**b**) G1T10PVDF (**c**) G2T10PVDF (**d**) G3T10PVDF (**e**) G4T10PVDF (**f**) G5T10PVDF (**g**) G4T20PVDF (**h**) G4T30PVDF.

AC conductivity (σ*_ac_*) of films calculated by using the relation σ*_ac_* = ɛ_o_ε′ωtanδ (angular frequency = ω = 2πf) σ*_ac_* of pure PVDF and nanocomposites plotted against frequency are shown in Figure 6A. σ*_ac_* rises when amount of conducting graphene is raised due to formation of conducting routes. σ*_ac_* is further raised when the ceramic concentration is raised. Among all nanocomposites, G4T20PVDF has the highest σ*_ac_*. Ceramic addition improves σ*_ac_* due to formation of a charge transport network within the nanocomposites. The results are in complete harmony with previously literature findings [31]. Comparative values of ε′, tanδ and σ*_ac_* of pure polymer, G4T10PVDF and G4T20PVDF nanocomposites at diverse frequencies are shown in Table 1. Electric modulus is used to explain transport of electrical charges within the dielectric medium. Real (M′) and imaginary parts (M″) of electric modulus were calculated by using relations; M′ = ε′/(ε″^2^ + ε′^2^) and M″ = ε″/(ε′^2^ + ε″^2^), respectively. M′ and M″ of pure polymer and all ternary nanocomposites plotted against log f are shown in Figure 6B,C. Figure 6B exhibits that M′ rises with rise in frequency till it acquires its maximum value, known as asymptotic value (M_∞_). Achieving M_∞_ is indicative of the capacitive nature of the ternary nanocomposites. It is also indicative of a relaxation process which extends over a long frequency range. Figure 6C shows that M″ also rises and reaches a maximum value, M″max, after which it starts to decline with rise in high frequency range [32]. At low frequency range charges and charge carriers are able to move freely according to applied electric field and may travel long distance till M″max is achieved. However at high frequencies, charges or charge carriers may move to small course only as they do not have sufficient time for alliance as there electric field is rapidly changing [33,34].

## 4. Conclusions

In summary, in this research work, we have demonstrated the successful fabrication of new flexible ternary nanocomposite films by combination of PVDF polymer used as base matrix and to achieve flexibility with titania and graphene additives providing their specific properties. The comprehensive characterization of prepared composites with different ratios of these three components was performed to confirm their morphologies, structures, functional groups, thermal stability and their dielectric properties. Results showed that nanocomposite G4T20PVDF (G:TiO_2_:PVDF = 4:20:100) exhibited a dielectric constant and energy loss of 70.4 and 0.39 at 100 Hz. Calculation of AC conductivity and electric modulus further confirmed that prepared nanocomposite films are effective dielectric materials which are proposed for use in the electronics industry for development of next generation flexible energy storage devices.

## Figures and Tables

**Table 1:** Dielectric permittivity (ε′), loss tangent (tanδ) and AC conductivity (σ*_ac_*) of neat PVDF, G4T10PVDF and G4T20PVDF.

Frequency (Hz)	Dielectric Permittivity (ε′)			Loss Tangent (tanδ)			AC Conductivity (σ*_ac_*) (Sm^−1^)		
	Neat PVDF	G4T10PVDF	G4T20PVDF	Neat PVDF	G4T10PVDF	G4T20PVDF	Neat PVDF	G4T10PVDF	G4T20PVDF
20	20.5	42.1	159.8	0.6	0.5	0.5	1.1 × 10^−8^	8.4 × 10^−9^	1.8 × 10^−8^
40	13.1	36.3	139.8	0.4	0.1	0.49	6.5 × 10^−9^	4.1 × 10^−9^	7.6 × 10^−8^
100	9.3	24.4	70.4	0.3	0.07	0.39	1.8 × 10^−8^	1.9 × 10^−8^	6.9 × 10^−8^
10^3^	7.6	22.9	47.7	0.07	0.03	0.21	6.8 × 10^−9^	3.5 × 10^−8^	5.7 × 10^−7^
10^4^	7.0	21.7	39.9	0.03	0.06	0.09	1.3 × 10^−7^	7.2 × 10^−7^	2.1 × 10^−6^
10^5^	6.8	19.2	36.8	0.02	0.17	0.05	7.1 × 10^−7^	1.7 × 10^−5^	9.6 × 10^−6^
10^6^	6.7	12.7	35.0	0.02	0.26	0.03	6.9 × 10^−6^	1.9 × 10^−5^	6.6 × 10^−5^
2 × 10^6^	6.7	6.7	11.6	34.8	0.02	0.25	0.03	1.5 × 10^−5^	3.5 × 10^−4^

## References

[1] Jung S., Albariqi M., Gruntz G., Al-Hathal T., Peinado A., Garcia-Caurel E., Nicolas Y., Toupance T., Bonnassieux Y., Horowitz G. (2016). A tips-tpdo-tetracn-based n-type organic field-effect transistor with a cross-linked pmma polymer gate dielectric. ACS Appl. Mater. Interfaces.

[2] Rajib M., Shuvo M.A.I., Karim H., Delfin D., Afrin S., Lin Y. (2015). Temperature influence on dielectric energy storage of nanocomposites. Ceram. Int..

[3] Huang B., Li M., Mei T., McCoul D., Qin S., Zhao Z., Zhao J. (2017). Wearable stretch sensors for motion measurement of the wrist joint based on dielectric elastomers. Sensors.

[4] Yao Z., Song Z., Hao H., Yu Z., Cao M., Zhang S., Lanagan M.T., Liu H. (2017). Homogeneous/inhomogeneous-structured dielectrics and their energy-storage performances. Adv. Mater..

[5] Yaqoob U., Chung G.-S. (2017). Effect of surface treated mwcnts and batio3 nanoparticles on the dielectric properties of a P(VDF-TrFe) matrix. J. Alloys Compd..

[6] Ishaq S., Kanwal F., Atiq S., Moussa M., Losic D. (2019). Synthesis of three phase graphene/titania/polydimethylsiloxane nanocomposite films and revealing their dielectric and impedance properties. Ceram. Int..

[7] Zhang L., Shan X., Bass P., Tong Y., Rolin T.D., Hill C.W., Brewer J.C., Tucker D.S., Cheng Z.-Y. (2016). Process and microstructure to achieve ultra-high dielectric constant in ceramic-polymer composites. Sci. Rep..

[8] Feng Y., Li M.-L., Li W.-L., Zhang T.-D., Zhao Y., Fei W.-D. (2018). Polymer/metal multi-layers structured composites: A route to high dielectric constant and suppressed dielectric loss. Appl. Phys. Lett..

[9] Zhao B., Hamidinejad M., Zhao C., Li R., Wang S., Kazemi Y., Park C.B. (2019). A versatile foaming platform to fabricate polymer/carbon composites with high dielectric permittivity and ultra-low dielectric loss. J. Mater. Chem. A.

[10] Pan Z., Yao L., Zhai J., Shen B., Wang H. (2017). Significantly improved dielectric properties and energy density of polymer nanocomposites via small loaded of batio3 nanotubes. Compos. Sci. Technol..

[11] Yang W., Yu S., Sun R., Du R. (2011). Nano- and microsize effect of ccto fillers on the dielectric behavior of ccto/pvdf composites. Acta Mater..

[12] Arbatti M., Shan X., Cheng Z.Y. (2007). Ceramic–polymer composites with high dielectric constant. Adv. Mater..

[13] Wang S., He X., Chen Q., Chen Y., He W., Zhou G., Zhang H., Jin X., Su X. (2018). Graphene-coated copper calcium titanate to improve dielectric performance of ppo-based composite. Mater. Lett..

[14] Qi F., Chen N., Wang Q. (2018). Dielectric and piezoelectric properties in selective laser sintered polyamide11/batio3/cnt ternary nanocomposites. Mater. Des..

[15] Wang D., Zhou T., Zha J.-W., Zhao J., Shi C.-Y., Dang Z.-M. (2013). Functionalized graphene–batio 3/ferroelectric polymer nanodielectric composites with high permittivity, low dielectric loss, and low percolation threshold. J. Mater. Chem. A.

[16] Chanmal C., Jog J. (2008). Dielectric relaxations in pvdf/batio3 nanocomposites. Express Polym. Lett..

[17] Zhang L., Wang Z., Xu C., Li Y., Gao J., Wang W., Liu Y. (2011). High strength graphene oxide/polyvinyl alcohol composite hydrogels. J. Mater. Chem..

[18] Grant F. (1959). Properties of rutile (titanium dioxide). Rev. Mod. Phys..

[19] Kovtyukhova N.I., Ollivier P.J., Martin B.R., Mallouk T.E., Chizhik S.A., Buzaneva E.V., Gorchinskiy A.D. (1999). Layer-by-layer assembly of ultrathin composite films from micron-sized graphite oxide sheets and polycations. Chem. Mater..

[20] Park S., An J., Potts J.R., Velamakanni A., Murali S., Ruoff R.S. (2011). Hydrazine-reduction of graphite-and graphene oxide. Carbon.

[21] Yaqoob U., Uddin A.I., Chung G.-S. (2016). The effect of reduced graphene oxide on the dielectric and ferroelectric properties of pvdf–batio 3 nanocomposites. RSC Adv..

[22] Masuda Y., Kato K. (2009). Synthesis and phase transformation of TiO_2_ nano-crystals in aqueous solutions. J. Ceram. Soc. Jpn..

[23] Zhang W., He Y., Zhang M., Yin Z., Chen Q. (2000). Raman scattering study on anatase TiO_2_ nanocrystals. J. Phys. D Appl. Phys..

[24] Li R., Chen C., Li J., Xu L., Xiao G., Yan D. (2014). A facile approach to superhydrophobic and superoleophilic graphene/polymer aerogels. J. Mater. Chem. A.

[25] Musić S., Gotić M., Ivanda M., Popović S., Turković A., Trojko R., Sekulić A., Furić K. (1997). Chemical and micro structural properties of tio2 synthesized by sol-gel procedure. Mater. Sci. Eng. B.

[26] Tantis I., Psarras G., Tasis D. (2012). Functionalized graphene–poly (vinyl alcohol) nanocomposites: Physical and dielectric properties. Express Polym. Lett..

[27] He F., Lau S., Chan H.L., Fan J. (2009). High dielectric permittivity and low percolation threshold in nanocomposites based on poly(vinylidene fluoride) and exfoliated graphite nanoplates. Adv. Mater..

[28] Kalini A., Gatos K., Karahaliou P., Georga S., Krontiras C., Psarras G. (2010). Probing the dielectric response of polyurethane/alumina nanocomposites. J. Polym. Sci. B Polym. Phys..

[29] Ping F., Lei W., Jintao Y., Feng C., Mingqiang Z. (2012). Graphene/poly(vinylidene fluoride) composites with high dielectric constant and low percolation threshold. Nanotechnology.

[30] Li J., Seok S.I., Chu B., Dogan F., Zhang Q., Wang Q. (2009). Nanocomposites of ferroelectric polymers with tio2 nanoparticles exhibiting significantly enhanced electrical energy density. Adv. Mater..

[31] Mo T.-C., Wang H.-W., Chen S.-Y., Yeh Y.-C. (2008). Synthesis and dielectric properties of polyaniline/titanium dioxide nanocomposites. Ceram. Int..

[32] Pradhan D., Samantaray B., Choudhary R., Thakur A. (2005). Complex impedance analysis of layered perovskite structure electroceramics—NaDyTiO_4_. J. Mater. Sci..

[33] Singh G., Tiwari V. (2009). Effect of zr concentration on conductivity behavior of (1−x) pmn-xpz ceramic: An impedance spectroscopy analysis. J. Appl. Phys..

[34] Amin M., Rafique H.M., Yousaf M., Ramay S.M., Atiq S. (2016). Structural and impedance spectroscopic analysis of sr/mn modified BiFeO_3_ multiferroics. J. Mater. Sci. Mater. Electron..

